# Despite good intentions, the regulation on in vitro diagnostic medical devices (IVDR) in Europe could impact negatively on preparedness and response for the next pandemic

**DOI:** 10.2807/1560-7917.ES.2026.31.2.2500553

**Published:** 2026-01-15

**Authors:** Richard Molenkamp, Lance D Presser, Sylvain Baize, Delphine Pannetier, Chantal BEM Reusken, Christian Drosten, Marion Koopmans

**Affiliations:** 1 Department of Viroscience, Erasmus MC, Rotterdam, The Netherlands; 2 Center for Infectious Disease Control, National Institute for Public Health and the Environment, Bilthoven, The Netherlands; 3 National Reference Center for Viral Hemorrhagic Fevers - Unit of Biology of Emerging Viral Infections, Institut Pasteur, Lyon, France; 4 National Reference Center for Viral Hemorrhagic Fevers, Laboratoire P4 Jean Mérieux INSERM, Lyon, France; 5 Section Virology, Division Infectious Diseases and Immunology, Department Biomolecular Health Sciences, Faculty Veterinary Medicine, Utrecht University, Utrecht, The Netherlands; 6 Institute of Virology, Charité Universitätsmedizin Berlin, Berlin, Germany

**Keywords:** In Vitro Diagnostics Regulations, IVDR, pandemic, preparedness, diagnostic test, Europe

## Abstract

Currently, there is concern and uncertainty in the European and North American markets for in vitro diagnostics regarding the regulation of in vitro diagnostic tests. In the European Union, starting from May 2022, the regulation on vitro diagnostic medical devices (IVDR) has replaced the directive on in vitro diagnostic medical devices (IVDD). The IVDR, while written with the good intentions to ensure patient safety and health while supporting innovation and transparency, has resulted in uncertainty, instances of disruption of diagnostic development, and concerns related to pandemic preparedness and response. We here outline the history, current situation and concerns regarding pandemic preparedness in Europe. Finally, we make recommendations that could improve the IVDR while supporting pandemic preparedness.

## Background

The development of in vitro diagnostic devices and tests (IVDs), whether developed by (private) manufacturers or within (public) health institutions, requires a concerted series of activities ranging from research to validation to production and implementation. Each of these activities is regulated by extensive quality control mechanisms through quality management systems and associated accreditation/certification. Stricter regulation of the market of IVDs in Europe has culminated in the implementation of the regulation on in vitro diagnostic medical devices (IVDR). This regulation came into force in May 2022 [1] and, in contrast to its predecessor the directive on in vitro diagnostic medical devices (IVDD), enforces implementation in European Union (EU) countries through EU law. Under the IVDD, manufacturers could self-certify the majority of their IVDs but under the IVDR, the majority of IVDs need to be Conformité Européenne (CE) certified by independent bodies. The development of this regulation was aligned with the medical device regulation (MDR) which was implemented following incidents with medical devices. Examples of such incidents with adverse effects on health were the *Poly Implant Prothèse* breast implants and the use of non-medical grade silicone as well as the metal-on-metal hip prostheses [2]. In line with the MDR, the purpose of the IVDR is to ensure a high level of patient safety and health while supporting innovation. Other expected benefits of the IVDR include increased transparency of IVD performance and, through for example additional requirements for monitoring of performance, improvements for post market surveillance and vigilance.

General concerns about the implementation of the IVDR have been voiced before [3-5]. Here we focus on several additional issues with consequences for public health that we see as potential unintended negative effects of the IVDR.

## The limitations of the exemption from IVDR for in-house IVDs (article 5.5): risk of losing expertise and infrastructure

Many medical and public health microbiology laboratories develop in-house in vitro diagnostics (IH-IVDs), especially in preparation for emerging diseases for which the market for diagnostics is challenging given the unpredictability of infectious disease outbreaks and the high risk of low or negative return on investment. The IVDR provides an exemption for IH-IVDs, provided that the laboratories fulfil several requirements (Table) and provide justification of the use of an IH-IVD over a CE-IVD by demonstrating that the target patient group's specific needs cannot be met at the appropriate level of performance by an equivalent CE-IVD available on the market. While this appears to be clear, this justification is nevertheless ambiguous, as it remains unclear which arguments for justification and equivalence are accepted. For instance, many CE-IVDs come with dedicated equipment while most IH-IVDs are designed for open platforms that allow for integration of workflows, flexibility and for scaling up when needed, reducing costs and allowing built-in outbreak preparedness and response for laboratories. Would this argument of flexibility and workflow integration be an acceptable justification for the use of an IH-IVD even if the CE-IVD is equivalent to the IH-IVD in every other aspect? In addition, important (proprietary) information from manufacturers of CE-IVDs that could be required for proper assessment of performance and equivalence by the customer, such as primer sequences for RT-PCR, is usually not available to that customer. 

**Table:** Article 5.5 requirements and date of enforcement during the progressive roll-out of the regulation on in vitro diagnostic medical devices

Description	Requirement	Date of enforcement
All IH-IVDs must comply with the general safety and performance requirements set out in Annex I of the IVDR.	Annex I	26 May 2022
IH-IVDs are not transferred to another legal entity.	5.5a	26 May 2022
Manufacture and use of the devices occur under appropriate quality management systems.	5.5b	26 May 2024
The laboratory is compliant with EN ISO 15189.	5.5c	26 May 2024
The laboratory justifies use of IH-IVD.	5.5d	26 May 2030
The laboratory provides all information to their competent authority.	5.5e	26 May 2024
The Laboratory draws up a public declaration of compliance.	5.5f	26 May 2024
Additional information on production of class D^a^ IH-IVDs is provided.	5.5g	26 May 2024
Manufacturing of class D IH-IVD conforms with article 5.5g.	5.5h	26 May 2024
Post-marketing surveillance of IH-IVDs is performed.	5.5i	26 May 2024
IH-IVDs are not manufactured on industrial scale.	5.5	26 May 2022

^a^ Class D devices belong to the highest risk class in relation to IVDR.

Development and use of IH-IVDs requires extensive quality control mechanisms such as validation, control and registration of reagents and determining boundaries or cut-offs for different types of controls. There is a profound risk that laboratories will increasingly switch to CE-IVD tests and that the availability of open platforms and the expertise to develop and implement IH-IVDs, will be gradually lost altogether.

## Direct and indirect consequences for pandemic preparedness and response: risk of considerable delays during outbreaks or pandemics

The barriers and risks that the IVDR imposes on laboratory systems are particularly apparent in the context of emerging infections and pandemic preparedness and response. The first months in an outbreak or pandemic are crucial for the public health response [6], as there is still opportunity to contain or slow down the outbreak. In this phase, the availability of an IVD relies on the expertise and work performed in reference/expert laboratories. For the COVID-19 pandemic (Figure), the first IH-IVDs were developed and validated by (consortia of) expert laboratories within just 2 weeks after the release of the genome sequence [7-9]. The protocols were published or made publicly available allowing implementation in a large number of reference/expert and routine laboratories in and outside Europe. As a result, testing for patient care and infectious disease control was available before the public health emergency of international concern (PHEIC) was declared. The first commercial IVDs became available on the EU market at the end of March 2020, almost 3 months after the start of the pandemic [10,11]. With the implementation of the IVDR and its increased regulatory requirements, it is expected that admission of IVDs on the EU market will now take considerably longer. This is not only true at the beginning of an outbreak/pandemic as the dynamics of a pandemic could lead to assay failures due to accumulation of mutations in the genome of the pathogen. This is not a hypothetical scenario as evidenced by the emergence of the severe acute respiratory syndrome coronavirus 2 (SARS-CoV-2) variants and their effect on certain IVDs [12] or the emergence of monkeypox virus clade Ib in central Africa in 2024 [13,14]. In an evolving pandemic it is crucial to monitor genome changes and to adapt, validate and implement improved IVDs continuously and rapidly. The regulatory framework of the IVDR is not well suited for this purpose. In addition, since the IVDR restricts the transfer of IH-IVDs to other legal entities, it interferes with the rapid sharing of reagents, again delaying public health response.

**Figure:**
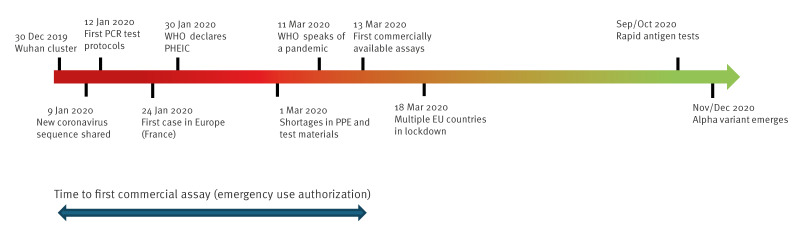
Timeline of main events related to in vitro diagnostics in the first year of the COVID-19 pandemic, 2020

EU: European Union; PHEIC: public health emergency of international concern; PPE: personal protective equipment; WHO: World Health Organization.

## Increased regulatory burden: risk of market failure and dependency

The increased requirements and regulatory tasks will place a disproportional burden on laboratories that are usually not compensated, and raise concerns of a potential increase in the costs of diagnostic services [15]. Similarly, the administrative burden could be disproportionate for small manufacturers of IVDs. These companies often provide niche products for rare diseases/pathogens that are not always commercially attractive. This could lead to the withdrawal of essential products from the EU market, a risk that has been acknowledged by the European Commission and has led to extension of transition periods and a gradual roll-out [16]. In the case of large (public) health threats, article 54 of the IVDR allows for a derogation in the interest of public health or patient safety/health. The article, however, only allows for derogation for a specific IVD (developed in-house by an expert laboratory or by a manufacturer before CE-IVD certification) and only upon a duly justified request of a competent authority to the European Commission and EU countries. Especially at the start of a pandemic or health threat, newly developed IVD are predominantly IH-IVDs and are usually implemented with slight modifications to align with the laboratory's routine procedures. They can therefore not be classified as a ‘specific IVD’. In addition, since this article only provides for derogation of one specific IVD per application, it severely limits the availability of alternatives. There is a risk of market failure as the demand for the exempted IVD could surpass the supply. If testing will be dependent on one specific IVD, the public health response could be severely impacted when this IVD is no longer suitable, in case of a change in the genome of the pathogen that would impact performance of the IVD. An example of negative effects of dependency of one provider of an IVD in an outbreak situation could be seen in the initial phase of the COVID-19 pandemic in the United States (US). Centrally organised distribution of PCR kits was hampered by contamination problems in these kits, delaying the implementation of large-scale COVID-19 testing. While similar contamination issues were observed in Europe [17], the response was less affected because of a decentralised implementation of tests, with reagents ordered by individual laboratories from different manufacturers. Finally, an important challenge here is that laboratory investments in assay development precede the declaration of public health threats, and decisions on potential derogation will invariably be too late when depending on an EU-level declaration.

## International context

The IVDR bears great similarities to the regulatory framework for diagnostics in the US. The American Society for Microbiology, representing diagnostic laboratories across the country, has expressed concern about the potential unintended negative impact of this regulatory framework for emerging disease preparedness [15]. For instance, the diagnostic response to the spread of avian influenza in cattle was delayed, leading to calls to reconsider this framework for preparedness and response [18]. The US regulatory framework is also endorsed for the African response to mpox by the Africa Centres for Disease Control and Prevention, and the potential for similar unintended negative impact of this regulatory framework has been noted. For instance, two commercial diagnostic platforms for mpox received emergency use authorisation more than 2 years after the first international PHEIC was declared [19], a situation that has also been noted for viral haemorrhagic fever diagnostics in that region. Historically, (European) expert laboratories have been at the front end of diagnostic preparedness and were among the first to develop widely applicable diagnostic tests for SARS-CoV-1, SARS-CoV-2, monkeypox virus clade Ib and other emerging pathogens. With full enforcement of the IVDR, this response would be severely hampered. 

## Perspective and recommendations

The IVDR has clear benefits. It requires manufacturers and laboratories to be more transparent about the nature and the performance of (IH-)IVDs. It requires that IVDs are more traceable, allowing direct action in case of incidents with an (IH-)IVD. Risk classification in the IVDR is now more logical and understandable. Clinical evidence and the compulsory post-market surveillance required from manufacturers of IVDs and from laboratories that develop IH-IVDs improves responsibility for the continuous quality and improvement of IVDs. It is expected that these provisions will increase the quality of IVDs on the EU market in general [1]. However, as we have argued above, IVDR poses many challenges for preparedness and response to emerging diseases and public health. With some modifications (Box) that align with a recent publication from the European Federation of Clinical Chemistry and Laboratory Medicine (EFLM) [20], the IVDR could still be turned into a strong and suitable instrument that takes into account the complexity and the need for rapid response in case of pandemic threats.

Box **Proposed changes to the in regulation on vitro diagnostic medical devices with respect to in-house in vitro diagnostics**

1. Relieve the requirement of compliance to Annex I but instead require accreditation to EN-ISO-15189 from laboratories that develop and use IH-IVDs. The quality of IH-IVDs in general is very high, especially in EN-ISO-15189 accredited laboratories [21]. Most of the relevant requirements of Annex I for IH-IVDs in infectious diseases are also mandatory in EN-ISO-15189. Accredited laboratories are visited periodically (usually yearly) and need to show compliance at every visit to keep their accreditation. Harmonisation of the IVDR with EN-ISO-15189 in this way would be efficient, effective, and would decrease double-oversight, confusion and administrative burden.
2. Relieve the requirement of the justification of use of an IH-IVD and the burden of proof at the laboratory end that the target patient group's specific needs cannot be met or cannot be met at the appropriate level of performance by an equivalent (CE-IVD) device available on the market. Laboratories that develop IH-IVDs employ professionals that are well aware of the impact that the quality of IVDs can have on patient safety and health. They act according to professional standards. Furthermore, the framework of the EN-ISO-15189 accreditation requires the continuous demonstration of competencies of staff and certifies that these professional standards are met. Important aspects of the IVDR such as providing clinical evidence, post-market surveillance and risk assessments of all procedures are an integral part of the EN-ISO-15189 accreditation. This would allow laboratories flexibility and preserve the expertise and innovation power that is most needed for preparedness and response to the next pandemic, or other unique clinical or public health situations.
3. Add a provision that, in crisis situations, would allow health institutions to distribute IVDs or reagents to other health institutions, being different legal entities.
4. For IH-IVDs, shift the focus of the IVDR from regulation to providing mechanisms to increase transparency and accountability for laboratories that develop and use these IH-IVDs. This could provide competent authorities with useful tools to manage potential incidents that may occur with the use of IVDs.

IH-IVDs: in-house in vitro diagnostics; IVD: in vitro diagnostics; IVDR: regulation on in vitro diagnostic medical devices.

## Conclusion

The IVDR was developed and is being implemented with the expectation that it would increase patient safety and health while maintaining or supporting innovation. However, especially for emerging infectious diseases, this regulation poses obstacles to preparedness and response. With some modifications proposed in this manuscript, the IVDR could be re-designed, retaining the initial intention of increased patient safety but also mitigating the major drawbacks that may decrease preparedness and response for emerging pathogens.

## Data Availability

Not applicable.
